# Cotard's syndrome and delayed diagnosis in Kashmir, India

**DOI:** 10.1186/1752-4458-2-1

**Published:** 2008-01-11

**Authors:** Zaid A Wani, Abdul W Khan, Aijaz A Baba, Hayat A Khan, Qurat-ul Ain Wani, Rayeesa Taploo

**Affiliations:** 1 Department of Psychiatry, SKIMS Medical College, Srinagar, Jammu and Kashmir, India; 2 Department of Surgery, Government Medical College, Srinagar, Jammu and Kashmir, India; 3 Department of Gynaecology and Obstetrics, Government Medical College, Srinagar, Jammu and Kashmir, India

## Abstract

Cotard's syndrome is a rare syndrome, characterized by the presence of nihilistic delusions. The syndrome is typically related to depression and is mostly found in middle-aged or older people. A few cases have been reported in young people with 90% of these being females. We present a case of a young pregnant woman suffering from Cotard's syndrome. This is the first report of this syndrome in a pregnant woman. The case was diagnosed late, due to lack of awareness of psychiatric problems in primary care physicians resulting in undue suffering, loss of precious time and resources for the patient. Besides highlighting the rare combination of pregnancy and Cotard's syndrome this report delineates the difficulties faced by patients with such symptoms in a low resource setting.

## Background

In 1880 Jules Cotard, a French psychiatrist described several patients who suffered from a syndrome referred to as *délire de negation *[1]. This rare syndrome is characterized by the presence of nihilistic delusions that one is dead or the world no longer exists [2]. The syndrome is typically related to depression and is usually encountered in middle-aged or older people. Advanced age has been found to increase the likelihood of developing Cotard's syndrome. Nevertheless, there are a few cases reported in the literature affecting young people with almost 90% of those affected being females [3-7]. We present a case of a pregnant woman suffering from Cotard's syndrome that was diagnosed late, due to lack of awareness of psychiatric symptoms and failure to recognise these by primary care physicians resulting in delayed treatment of a potentially treatable condition.

## Case presentation

Mrs. S is a 28 year old married female who is an illiterate housewife. This patient came to our outpatient department with symptoms of decreased sleep and appetite and lack of interest in her usual household chores. The patient complained that her liver was "putrefying" and her heart was "altogether absent". She also reported that when she walked she could not feel her body and reported that her "stomach was missing". She was 6 months into a pregnancy with her first child.

Her problems had started after she had left her joint family subsequent to frequent quarrels with her in-laws and had started to live along with her husband in a small rented accommodation. Initially she developed loss of sleep and palpitations and a feeling of loneliness which progressed to loss of interest in her surroundings and her daily household work. She began to have feelings of guilt about herself and felt that she had done something wrong in leaving the joint family. The patient's husband took her to a doctor who described her symptoms as 'vague', diagnosed her as having 'weakness' and prescribed multivitamin tablets. Over the next 6 weeks the patient's symptoms failed to improve. At that point, she consulted another doctor who, after an initial two weeks of unsuccessful treatment for her 'vague' abdominal complaints (missing stomach and putrefying liver), referred her for a surgical consultation. After examining the patient and carrying out various investigations, including ultrasonography and endoscopy, the surgeon continued the previous treatment and added an antibiotic for a week. Within the next 2 weeks the patient visited a faith healer and a practitioner of the Indian system of medicine. After this the patient consulted yet another doctor who referred her for a psychiatric consultation. In the meantime 3 months had passed since she had developed her symptoms. She had previously made an unsuccessful attempt at suicide by taking organophosphorus poison for which treatment included medications and observation. She had been advised to seek psychiatric consultation on discharge but had not complied. One month after the suicide attempt she had again thought of suicide but did not act on these thoughts because of her "fear of God".

The patient was dressed appropriately and was well groomed. Her speech was coherent and relevant. Her complaints included insomnia, a general loss of interest, lack of concentration and a feeling of hopelessness. She was found tohave depressed mood but would occasionally smile while answering questions related to her symptoms because the "doctors wouldn't believe her". She had partial insight into her symptoms. She was not concerned about her pregnancy and had ambivalent feelings of love and hate towards the unborn child. There was no disorder of content or possession of thought. Physical examination was unremarkable except the patient was anaemic with haemoglobin of 8.5 mg/dl. Other routine investigations, including thyroid function tests, were normal. A CT scan or an MRI was advised by a consultant to rule out any organic problem. The CT was not done because of her pregnancy and the MRI could not be done because of financial reasons. A DSM IV [8] diagnosis of Major Depressive Disorder with Nihilistic Delusions was made.

ECT was suggested but the patient refused because of the social stigma associated with the procedure. Other possibilities were explored. Initially, the patient was reluctant to take drugs because of her pregnancy. After persistence and a few counselling sessions the patient agreed. The patient could not tolerate fluoxetine 20 mg because of persistent nausea. Escitalopram caused similar problems. Both drugs were discontinued within a week. Tricyclic antidepressants were not prescribed because of the side effects. The patient was put on mirtazapine 15 mg and haloperidol 5 mg. Both of these were increased to 30 mg and 10 mg, respectively, over the next five days. The patient showed remarkable recovery within the next 4 weeks. MADRAS [9] scores came down to 18 from an initial score of 44. Over weeks four to eight the patient's mood and delusions improved. The improvement was sustained for four months and, at the time of writing, was in remission.

## Discussion

Cotard's syndrome is a rare syndrome which presents with severe nihilistic delusions. Treating severe nihilistic depression in a pregnant woman can be a dilemma both morally and clinically. The various factors which are to be kept in mind include the care of the foetus and the choice of an effective drug for the patient. Pharmacotherapy for depression during pregnancy requires an assessment of the risks and benefits of treatment for both mother and foetus. The risks of treatment should be compared with the risks of not treating depression. In our patient the presence of depression with severe nihilistic delusions necessitated treatment for better foetal and maternal outcomes [10].

Electroconvulsive therapy has been reported to be relatively safe in pregnant women with severe, refractory depression [11]. ECT has been found beneficial in Cotard's syndrome [12,13] so we recommended it to our patient to reduce the risk of suicide and to prevent harm to the foetus. The patient's reluctance to take drugs demonstrated her concern for the foetus. After intolerance to two SSRIs, the patient was given Mirtazapine as it has not been reported to have adverse foetal effects [14]. Haloperidol was added for psychotic features as it has also been found to be safe choice in pregnancy [15].

This is one of few reported cases of Cotard's syndrome in a young female patient. Most of the cases have been reported in elderly patients [1].

The importance of this case, apart from the rarity of this syndrome in young people, is that it highlights the problems faced by a patient where psychiatric symptoms were 'laughed at' and symptoms were described as 'vague' in the primary health care setting. This is mainly due to clinical bias as a result of unfamiliarity with psychiatric symptomatology. These problems lead to loss of precious time and resources for the patient and the community. With a proper referral in the first instance the patient could have prevented drain on her financial resources due to inappropriate investigations and consultations. This is important in this part of the world as a majority of people visiting hospitals are not covered with health insurance, and patients must pay from their own pocket for investigations, which can be very significant considering the economic conditions of the population where the per capita income is low. A prompt initial referral would have saved time, suffering and the long list of repeated investigations which including several complete blood counts, liver and kidney function tests, ultrasonographies and an endoscopy. Besides this, her cynicism which was palpable later could have been avoided.

Health professionals in primary care have difficulties in recognizing and diagnosing psychiatric disorders [16,17]. The main reason for the limited awareness among primary care physicians and medical specialists in our setting is insufficient psychiatric education during basic training. A five and half year course has just 30 days of training in psychiatry followed by an optional posting period of 2 weeks during internship. The problem is compounded by the relative lack of qualified psychiatrists working in the health system of Kashmir and inadequate support for primary care physicians. Time constraints on the part of the doctor can be an important factor as patients with psychiatric disorders take more time than other patients. Since the majority of the health care delivery systems in Kashmir are run by the government, and a doctor in a government hospital is supposed to see 50–60 patients in a three to four hour period, it is impossible to give adequate time to each patient, leading to faulty diagnosis and treatment, particularly of psychiatric disorders. One of the important factors which is important in our context is the virtually nonexistent two-way referral system. The 20 years of armed conflict in Kashmir has resulted in the failure of institutions, lack of accountability and subsequent severe mismanagement and collapse of health care delivery systems.

Improvements in diagnosis of psychiatric disorders by primary care physicians and medical specialists may be achieved with short training courses and updates in psychiatry, and particularly through frequent consultation liaison visits [18,19]. The number of days of posting in psychiatry for medical interns can be increased. There is a particular need to establish functioning two-way referral systems. This is particularly important in our setting where patients may not present with classical symptoms of psychiatric disorders.

## Conclusion

Cotard's syndrome is rare, and still rarer in young people. To the best of our knowledge this is first reported case in young pregnant woman. Delayed diagnosis due to unfamiliarity with psychiatric symptoms in primary care resulted in undue distress to the patient, unnecessary expense, and delay in providing effective treatment. Increasing exposure to psychiatric problems through short courses, consultation liaison psychiatry and through increased posting days in psychiatry in medical school may help to improve diagnosis in primary care.

## Competing interests

The author(s) declare that they have no competing interests.

## Authors' contributions

ZAW is the principal author of the paper. AWK revised and wrote a part of discussion. AAB revised and edited the document. HAK, QAW, RT collected the data, formatted the, manuscript and under took a final revision. All authors read and approved the manuscript.
